# Metabolism of hemicelluloses by root-associated *Bacteroidota* species

**DOI:** 10.1093/ismejo/wraf022

**Published:** 2025-02-06

**Authors:** Hannah Martin, Lucy A Rogers, Laila Moushtaq, Amanda A Brindley, Polly Forbes, Amy R Quinton, Andrew R J Murphy, Helen Hipperson, Tim J Daniell, Didier Ndeh, Sam Amsbury, Andrew Hitchcock, Ian D E A Lidbury

**Affiliations:** Molecular Microbiology - Biochemistry and Disease, School of Biosciences, The University of Sheffield, Western Bank, Sheffield, S10 2TN, South Yorkshire, United Kingdom; Molecular Microbiology - Biochemistry and Disease, School of Biosciences, The University of Sheffield, Western Bank, Sheffield, S10 2TN, South Yorkshire, United Kingdom; Molecular Microbiology - Biochemistry and Disease, School of Biosciences, The University of Sheffield, Western Bank, Sheffield, S10 2TN, South Yorkshire, United Kingdom; Plants, Photosynthesis and Soil, School of Biosciences, The University of Sheffield, Western Bank, Sheffield, S10 2TN, South Yorkshire, United Kingdom; Molecular Microbiology - Biochemistry and Disease, School of Biosciences, The University of Sheffield, Western Bank, Sheffield, S10 2TN, South Yorkshire, United Kingdom; Molecular Microbiology - Biochemistry and Disease, School of Biosciences, The University of Sheffield, Western Bank, Sheffield, S10 2TN, South Yorkshire, United Kingdom; School of Life Sciences, The University of Warwick, Gibbet Hill Road, Coventry, CV4 7AL, West Midlands, United Kingdom; Plants, Photosynthesis and Soil, School of Biosciences, The University of Sheffield, Western Bank, Sheffield, S10 2TN, South Yorkshire, United Kingdom; Plants, Photosynthesis and Soil, School of Biosciences, The University of Sheffield, Western Bank, Sheffield, S10 2TN, South Yorkshire, United Kingdom; School of Life Sciences, University of Dundee, Dow Street, Dundee, DD1 5EH, Scotland, United Kingdom; Plants, Photosynthesis and Soil, School of Biosciences, The University of Sheffield, Western Bank, Sheffield, S10 2TN, South Yorkshire, United Kingdom; Molecular Microbiology - Biochemistry and Disease, School of Biosciences, The University of Sheffield, Western Bank, Sheffield, S10 2TN, South Yorkshire, United Kingdom; Plants, Photosynthesis and Soil, School of Biosciences, The University of Sheffield, Western Bank, Sheffield, S10 2TN, South Yorkshire, United Kingdom; Molecular Microbiology - Biochemistry and Disease, School of Biosciences, The University of Sheffield, Western Bank, Sheffield, S10 2TN, South Yorkshire, United Kingdom

**Keywords:** Rhizosphere, Plant microbiome, *Flavobacterium*, *Bacteroidota*, Glycobiology, Root exudates, Polysaccharide utilisation loci

## Abstract

*Bacteroidota* species are enriched in the plant microbiome and provide several beneficial functions for their host, including disease suppression. Determining the mechanisms that enable bacteroidota to colonise plant roots may therefore provide opportunities for enhancing crop production through microbiome engineering. By focusing on nutrient acquisition mechanisms, we discovered *Bacteroidota* species lack high affinity ATP-binding cassette transporters common in other plant-associated bacteria for capturing simple carbon exudates. Instead, bacteroidota possess TonB-dependent transporters predicted to import glycans produced by plant polysaccharide breakdown. Metatranscriptomics (oat rhizosphere) identified several TonB-dependent transporters genes that were highly expressed in *Flavobacterium* (phylum *Bacteroidota*). Using *Flavobacterium johnsoniae* as the model*,* we experimentally validated the function of one highly expressed TonB-dependent transporter, identifying a conserved Xyloglucan utilisation loci conferring the ability to import and degrade xyloglucan, the major hemicellulose secreted from plant roots. Xyloglucan utilisation loci harbour an endoxyloglucanase related to family 5 subfamily 4 subclade 2D glycoside hydrolases carrying a mutation that we demonstrate is required for full activity towards xyloglucan. Based on analysing 700 soil metagenomes, subclade 2D glycoside hydrolases have radiated in soil and are prevalent among plant-associated bacteroidota and certain taxa affiliated with *Gammaproteobacteria*. In bacteroidota, particularly *Flavobacterium* species, xyloglucan utilisation loci organisation was highly conserved, which may increase their competitive ability to utilise xyloglucan. Given bacteroidota lack high-affinity nutrient transporters for simple carbon, instead possessing xyloglucan utilisation loci and similar gene clusters, our data suggests hemicellulose exudates provide them with an important carbon source in the rhizosphere.

## Introduction

Plants provide soils with the “fresh” carbon (C) required to support microbial growth, generating “hotspots” of activity in regions of C deposition, such as the rhizosphere [1, 2]. Microbial processing of plant-derived C has major consequences for the global terrestrial C cycle [3]. Each year, soil respiration (CO_2_ release) releases 10-15x more C than that emitted from anthropogenic activities [4]. Any change in the balance of production (C storage) versus respiration in response to global change will have significant ramifications for the climate [3]. Plant-derived C is partitioned into two major fractions: 1) structurally simple C (e.g. organic acids, amino acids, amines, and mono- and di-saccharides) and 2) structurally complex C (e.g. polysaccharides), which are believed to persist for longer [5]. Complex C can interact with the soil matrix and sometimes escapes microbial attack, directly contributing to C storage [5, 6]. In addition to biogeochemical cycling, nutrient inputs have a significant influence on plant microbiome assemblage and community structure [7], evidenced through the impact of crop domestication [8, 9].

Plant polysaccharides are major components of plant biomass, of which hemicelluloses, such as xyloglucan (XyG), typically constitute up to 50% [10]. In monocots (e.g. barley), cell wall XyG levels are much lower (2–10%) [11, 12]. XyG is secreted as root mucilage exudate in many angiosperms, including major cereal crops (e.g. barley), tomato, oilseed rape, and soybean [6, 13, 14]. Although XyG forms a minor component of cell wall biomass in cereal crops, this hemicellulose is still secreted at levels comparable to other plants [6, 13]. XyG secretion at the root tip and along the entire root axes contributes towards rhizosheath formation, a glycan-rich region protecting roots from abrasion, desiccation, and oomycete pathogen attack [6, 14–16]. Historically, mycorrhizal and saprophytic fungi were considered the major plant polysaccharide degraders, however, soil bacteria are emerging as integral players in their breakdown [17]. In forest and agricultural soil, plant detritus (leaf litter, cellulose) enriches members of the phyla *Pseudomonadota*, *Actinomycetota*, and *Bacteroidota* [18, 19]. XyG utilisation in the plant pathogen *Xanthomonas* is considered a virulence factor, enabling the bacterium to enter leaf cells [20], whereas *Cellvibrio* can breakdown this hemicellulose for growth [21, 22]. However, the consequences of polysaccharide exudation on plant-microbe interactions and root-microbiota assemblage are understudied.

Polysaccharide degradation requires specialised gene sets encoding carbohydrate-active enzymes (CAZymes) to initiate degradation [5, 17, 23]. CAZymes are categorised into broad functional groups, i.e., glycosyl hydrolase (GH) and carbohydrate esterase (CE) and are incredibly diverse (e.g. ~200 GH families), reflecting the enormous variety of carbohydrates found in nature. In bacteroidota, these gene sets are typically colocalised into discrete operons referred to as Polysaccharide Utilisation Loci (PUL) with bioinformatic prediction rapidly outpacing experimental validation of their function [23–26]. PUL are a hallmark of the phylum *Bacteroidota*, a deep branching group of Gram-negative bacteria that specialise in complex polymer degradation [23]. Through the efficient capture of glycans released from polysaccharide hydrolysis, PUL provide a competitive advantage for bacteroidota in gut and marine polysaccharide-rich environments, such as the human gut or leaf litter [5]. Despite these findings, the contribution of soil bacteroidota towards hemicellulose degradation in the plant microbiome is limited [27–29].

*Bacteroidota* spp. are enriched in numerous wild and domesticated plant microbiomes relative to the surrounding bulk soil [30–34]. The relative metabolic active of bacteroidota in the root endosphere and rhizosphere often exceeds that of its abundance [2, 34]. Significant interest in *Bacteroidota* spp. has emerged due to their role as indicators of good soil health [25] and suppressing various fungal and bacterial plant pathogens [35–40]. Recently, we discovered *Flavobacterium*, a genus within *Bacteroidota*, have adapted to life in the plant microbiome by specialising in organophosphorus utilisation and likely play a key role in increasing phosphate availability for plants [41–43]. The mechanisms underpinning their success in the plant microbiome, particularly root-associated communities, remain unknown. Re-analysis of the same proteomics dataset identified several CAZymes that are candidates for plant polysaccharide utilisation, suggesting that complex C utilisation represents a key lifestyle strategy for these bacteria [44].

In this study, we aimed to determine the mechanisms enabling the enrichment of taxa affiliated with *Bacteroidota* in the plant microbiome. Based on their relatives in marine and gut microbiomes, we hypothesised specialisation in polysaccharide utilisation is a niche-adapted trait driving this enrichment. By combining comparative (meta)genomics, metatranscriptomics, reverse genetics, and protein biochemistry, we reveal plant-associated bacteroidota likely specialise in utilising hemicelluloses found at the root-soil interface.

## Materials and methods

### Bacterial strains and growth medium

*F. johnsoniae* UW101 (DSM2064) was purchased from the Deutsche Sammlung von Mikroorganismen und Zellkulturen (DSMZ) collection. *Flavobacterium* sp. OSR005 and *Flavobacterium* sp. OSR003 were isolated previously [43]. *Flavobacterium* sp. F52 was kindly donated from the Cytryn lab [45]. *Flavobacterium* strains were routinely maintained on casitone yeast extract medium (CYE) [46] containing casitone (4 g L^−1^), yeast extract (1.25 g L^−1^), MgCl_2_ (350 mg L^−1^), and agar (20 g L^−1^). For conjugation experiments, MgCl_2_ was substituted with CaCl_2_ (1.36 g L^−1^). For growth experiments investigating hemicellulose degradation, *Flavobacterium* strains were grown in a modified minimal A medium [42, 43] containing NaCl (200 mg L^−1^), NH_4_Cl (450 mg L^−1^), CaCl_2_ (200 mg L^−1^), KCl (300 mg L^−1^), and MgSO_4_ (350 mg L^−1^). After autoclaving, filter-sterilised yeast extract (10 mg L^−1^), FeCl_2_ (2 mg L^−1^), MnCl_2_ (2 mg L^−1^), NaH_2_PO_4_ (100 mg L^−1^), and 20 mM HEPES buffer pH 7.4 were added. This medium was supplemented with either 0.25–0.4% (w/v) glucose, tamarind XyG (Megazyme, CAS Number: 37294–28-3), Xyloglucan oligosaccharides (hepta+octa+nona saccharides, Megazyme, CAS Number: 121591–98-8), or Carob galactomannan (Megazyme, CAS Number: 11078–30-1) as the sole C source. Growth assays were performed in 200 μL microcosms and incubated in a TECAN SPARK microtiter plate reader at 28°C using optical density measured at 600 nm (OD_600_). For soil enrichments, please see Fig. S1 for experimental details.

### Comparative proteomics of *Flavobacterium* species

Methods adapted from [43, 47] were combined. Please see supplementary methods for full details. Briefly, mid-late growth phase cells (n = 3) were lysed by boiling in lithium dodecyl sulfate (LDS) buffer (Expedeon) prior to SDS-PAGE. A single gel band containing all protein was excised, de-stained, washed, and digested (tryptic) prior to analysis by nanoLC-ESI-MS/MS. Protein sequence database (*F. johnsoniae* UW101, UP000214645, *Flavobacterium* sp. OSR005 (IMG genome (Taxon) ID: 8103301142) were used for MaxQuant and quantification was achieved using Label Free Quantification (LFQ). Statistical analysis and data visualisation was performed in Perseus [48]. The mass spectrometry proteomics data have been deposited to the ProteomeXchange Consortium via the PRIDE partner repository with the dataset identifier PXD053370.

### Bacterial genetics

To construct various Xyloglucan Utilisation Loci (XyGUL) mutants, the method from [49] was adapted, as per our previous study [42]. A full list of primers can be found in Table S1. Briefly, constructed plasmids were transformed into the donor strain *E. coli* S17–1 λpir (S17–1 λpir) and mobilised into *F. johnsoniae* via conjugation. To generate a clean mutation, counterselection using CYE containing 10% (w/v) sucrose was performed. For complementation of the *F. johnsoniae* mutants, complete genes and the 300-bp upstream region were cloned into replicative plasmid pCP11 and mobilised as before. Please refer to the supplementary methods for full details.

### Production and purification of recombinant GH5_4 homologs

Genes encoding the GH5_4 homologs (Fjoh_0774, BACOVA_02653, OSR005_04227 and OSR005_03871) lacking the N-terminal signal peptide and stop codon were amplified by PCR and ligated into the NdeI and XhoI sites of pET21a. Site-directed mutagenesis of the Trp252 residue in *Bo*GH5A was performed using the QuikChange II Site Directed Mutagenesis (SDM) Kit (Agilent Technologies) according to the manufacturer’s protocol.

For production of recombinant proteins, *E. coli* BL21 (DE3) transformed with the desired plasmid. Gene expression was induced when the culture reach OD_600_ ~0.6 using 0.4 mM (final concentration) IPTG and cells were incubated at 18°C overnight. Proteins were purified by immobilized metal affinity chromatography and size-exclusion chromatography. Please refer to supplementary methods for full details.

### Enzymatic assays of recombinant glycoside hydrolases

Purified recombinant GH5_4 homologs were screened for enzyme activity using the 3,5-dinitrosalicylic acid assay (DNSA) method [50]. Briefly, for enzyme kinetics 10–250 nM purified recombinant protein (n = 3) was incubated with decreasing concentrations (starting from 8 mg mL^−1^) of XyG. At each time point a subsample was taken and mixed with a stop solution (DNSA containing 10 mg ml^−1^ glucose), prior to boiling at 95°C for 15 min to develop the colour. To calculate the initial maximum velocity of the reaction (*V*_o_), at least five measurements were taken within the linear kinetics range (Fig. S7). Absorbance at 575 nm (A575) was quantified. A standard curve (n = 3) against known concentrations of glucose was used to convert A575 to the amount of freely available reducing ends produced during cleavage of the beta-glucan backbone of XyG. All assays were typically repeated with two separate batches of protein. For screening the promiscuous activity of OSR005–1, OSR005–2, *Bo*GH5A, *Bo*W252A, or *Bo*W252G, 1 μM of protein was incubated with 4 mg mL^−1^ polysaccharide for 30 min.

### Comparative genomics and metagenomics

The online platform IMG/JGI [51] was used to conduct most comparative genomics and RNA Seq analyses described in this study. For metatranscriptomics, the RNA Seq study associated with the *Avena fatua* rhizosphere microbial communities from Hopland, California, USA, for root-enhanced decomposition of organic matter studies was scrutinsed. In this study, RNA Seq data was mapped to 31 bacterial RNA reference datasets (genomes). The normalised read coverage values provided by the IMG pipeline were used as a proxy for relative gene expression. Table S2 provides a list of pfam domains and genomes used to perform a “function search” to obtain counts of three major bacterial transporter families; major facilitator superfamily, ABC transporters, and TonB-dependent transporters, including a subset associated with *Bacteroidota*-specific glycan importers that contain a surface exposed lipoprotein domain. Genomes and metagenomes were stored in genome sets (detailed in Tables S3 and S4), and BLASTP searches (E-value e^−40^) were performed using the “jobs function” using either Fjoh_0774 or a homolog (IMG gene id; 2 644 426 200) of the commercial recombinant endoxyloglucanase (GH5_4) from *Paenibacillus* sp. (Megazyme, CAS - 76 901-10-5). The latter was used as it represented a sequence from outside GH5_4 subclade 2. For metagenomes, retrieved open reading frames (ORFs, n = 7136) were locally aligned (BLASTP) against all GH5 ORFs (n = 1246) deposited in the CAZy database (CazyDB) [25]. The estimated gene copy index (calculated by using the average read coverage depth across a given contig) provided by IMG/JGI was used to calculate the relative abundance for each retrieved ORF. To aid with the identification and organisation of PUL, the PULDB online platform was also utilised [26]. Please refer to supplementary methods for details relating to 16S rRNA gene profiling of soil enrichments.

## Results

### Plant-associated bacteroidota induce distinct membrane transporters in the rhizosphere

**Figure 1:**
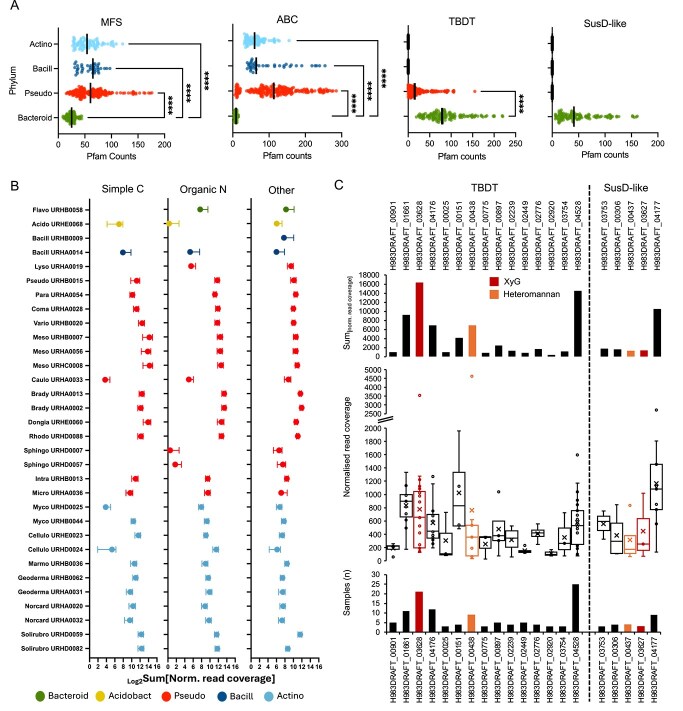
**Nutrient transporter diversity and *in planta* expression among plant-associated bacteria.** (**A**) Counts of four transporter families, based on pfam domains, identified within the genomes of plant-associated bacteria related to the four major phyla: *Actinomycetota*, *Bacilliota*, *Pseudomonoadota*, *Bacteroidota*. Asterisks denote significant differences in the mean number of individual transporter families found in between each phylum. Abbreviations: MFS, major facilitator superfamily; ABC, substrate binding domain of ATP-binding cassette transporters; TBDT, transmembrane TonB-dependent transporter; SusD-like, surface exposed ligand binding lipoprotein component of TonB-dependent transporters. (**B**) The average transcription levels (normalised read coverage) of SBPs expressed in 31 bacterial species relating to simple C, organic nitrogen, and all other functions (e.g. iron and phosphorus transport) across 47 oat rhizosphere and bulk soil metatranscriptomes. (**C**) TonB-dependent transporter (TBDT) and SusD-like expression in *Flavobacterium* sp. URHB0058 across the 47 metatranscriptomes. The sum of transporter expression across all samples (top panel), the number of samples each was detected in across the study (bottom panel), and the distribution of expression in detected samples (middle panel) is provided. TBDT-SusD-like components found in PUL predicted to capture XyG or heteromannan are highlighted.

Using the IMG/JGI database, we scrutinised the genomes (n = 492) of plant-associated bacteria related to the four major phyla, *Pseudomonadota*, *Bacillota*, *Actinomycetota*, *Bacteroidota* (Table S2). As queries, we used pfam domains associated with either the major facilitator superfamily (MFS) of permeases, substrate binding proteins (SBPs) of ABC-transporters primarily related to C and organic nitrogen acquisition, the transmembrane component of TonB-dependent transporters (TBDTs), or the surface-exposed outer membrane ligand binding lipoprotein component (SusD-like) of TBDTs (Table S2). There was a significant reduction of MFS- and SBP-related transporters in the genomes related to *Bacteroidota*, compared with all other three phyla (Fig. 1A). This included an omission of SBPs related to monosaccharides. In contrast, no TBDT genes were detected in the genomes of taxa affiliated with *Bacillota* or *Actinomycetota*, and *Bacteroidota* possessed significantly more TBDT genes than *Pseudomonadota* spp. Consistent with the literature [23, 52], only bacteroidota harbour genes encoding SusD-like components of TBDTs.

We analysed RNA Seq datasets affiliated with the *Avena fatua* (oat) rhizosphere metatranscriptomes deposited in IMG/JGI, which include in situ gene expression data for 34 bacterial genomes, including one *Flavobacterium* sp. (URHB0058). Taxa affiliated with *Pseudomonadota*, *Bacillota*, and *Actinomycetota* representatives all expressed SBP genes required for the high-affinity uptake of simple C and other small organic nitrogen compounds (Fig. 1B). For *Flavobacterium*, TBDT-SusD-like complexes were our focus as they represent markers for studying in situ gene expression of marine bacteroidota [53]. URHB0058 encodes 53 TBDTs of which 32 are paired with the SusD-like lipoproteins. Based on PULDB [26], 30 TBDT-SusD-like pairs are located within predicted PUL. Across the 47 metatranscriptomes, transcripts for only 15 TBDTs and five SusD-like lipoproteins were detected, of which four of the latter corresponded to one of the expressed TBDT genes (Fig. 1C). Two out of the four identified TBDT-SusD pairs (orange and red in Fig. 1C) were in PUL3 and PUL23 (numbers in PULDB) that we previously suggested are required for heteromannan and XyG utilisation, respectively [44]. The gene encoding a TBDT (H983DRAFT_03628) located in PUL 23 was one of the most highly expressed systems across all samples (Fig. 1C). Transcripts for other components of PUL3 and PUL23 were also detected across samples.

Based on a lack of ABC transporters and the in situ expression of TBDT genes, we hypothesised that plant-associated bacteroidota may have a diminished ability to compete for simple C root exudates and specialise in growing on more complex C, specifically hemicellulose polysaccharides. To test this hypothesis, we performed a simple enrichment whereby grassland soil microbial communities were supplemented with either simple (glucose, succinate) or complex C (XyG, galactomannan, and wheat arabinoxylan). After enrichment with complex C, based on 16S rRNA gene profiling, members of the *Bacteroidota* (*Flavobacterium* and *Cytophagaceae*) become the dominant members (65.2%) of the community, whereas taxa affiliated with *Gammaproteobacteria* dominated (76%) communities enriched with simple C (Fig. S1).

### Plant-associated *Flavobacterium* species possess a hybrid XyGUL

**Figure 2:**
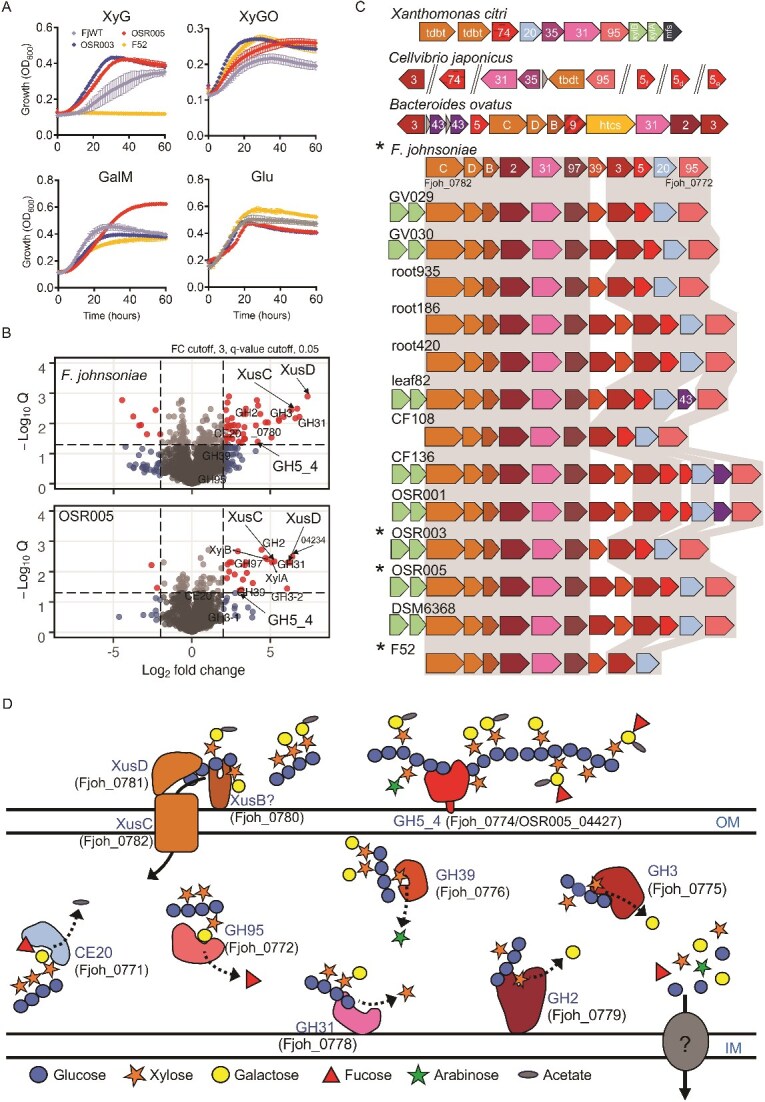
**Xyloglucan utilisation by soil *Bacteroidota*.** (**A**) *F. johnsoniae* grown on plant-associated *Flavobacterium* isolated from various crop rhizospheres were grown on either glucose (Glu), galactomannan (GalM), xyloglucan (XyG) hemicelluloses, or xylooligos (XyGOs) as the sole C and energy source. Data represent the mean of triplicate cultures and error bars denote standard deviation. (**B**) Proteins enriched in the whole-cell proteomes (n = 3) of either *F. johnsoniae* or *Flavobacterium* sp. OSR005 when grown on XyG compared to growth on glucose. Statistically significant (FDR-corrected *P* < 0.05) proteins with greater than 2-fold enrichment are highlighted. Proteins in the predicted XyGUL are labelled. (**C**) XyGUL share modules from *X. citri* and *B. ovatus* and are highly conserved among plant-associated *Flavobacterium* spp. (strain identifier labelled). (**D**) The predicted function and localisation of proteins encoded in the induced XyGUL with locus tags for *F. johnsoniae* provided. Colours in c and d represent the corresponding open reading frames and proteins. Locus tags correspond to *F. johnsoniae.* Numbers in d correspond to the predicted glycoside hydrolase family in the CAZY database. Asterisks represent the strain used in panel A. Abbreviations: OM, outer membrane; IM, inner membrane.

To determine if PUL 23 is involved in XyG utilisation we used *Flavobacterium johnsoniae* as the model, as this bacterium was previously reported to grow on hemicelluloses [54]. In addition, *Flavobacterium* sp. F52, and two *Flavobacterium* spp. (OSR005 and OSR003), isolated from the rhizosphere of oilseed rape [37], were also screened for their ability to grow on XyG, xylo-oligosaccharides (XyGO), and carob galactomannan (GalM). *F. johnsoniae*, OSR005, and OSR003 and grew well on XyG, with the latter two growing at a rate comparable to that on glucose (Fig. 2A). Despite not growing on XyG, *Flavobacterium* sp. F52 did grow on GalM and XyGO, as did the other three strains.

Whole-cell comparative proteomic analyses of *F. johnsoniae* and OSR005 grown on either XyG or glucose revealed the significant (FDR corrected P < 0.05, log_2_ FC > 2) enrichment of 68 and 74 proteins, respectively (Tables S5 and S6, Fig. 2B). Among the most differentially synthesised proteins was a PUL matching PUL 23 in *Flavobacterium* sp. URHB0058, hereafter named XyGUL. In *F. johnsoniae* and OSR005, XyGUL are encoded by Fjoh_0772–0782 and OSR005_04225–04238, respectively, and both contain a predicted endoxyloglucanase related to the GH5_4 family (Fjoh_0774; OSR005_4227) (Table 1). Both Fjoh_0774 and OSR005_4227 showed greater sequence homology to the *C. japonicus* GH5_4 [22] (*Cj*GH5D, encoded by *cel5D*) than to the *Bacteroides ovatus* GH5_4 [55] (*Bo*GH5A, encoded by BACOVA_02653) (Table 1, Fig. S2). *Cj*GH5D and *Bo*GH5A function as outer membrane-anchored XyG-specific 1–4-β-endoglucanases and are required to initiate degradation of the polysaccharide. The ORF encoding this XyG-specific 1–4-β-endoglucanase was absent from *Flavobacterium* sp. F52 despite harbouring an otherwise complete XyGUL (Fig. 2C).

**Table 1:** Glycoside hydrolase (GH5_4) homologs used in this study, including those subjected to site directed mutagenesis.

**Enzyme name**	**Host strain**	**Locus tag**	**Residue (position 252)**	**Clade**	**Subclade**
*Fj*GH5	*Flavobacterium johnsoniae*	Fjoh_0774	W (Trp)	2D	I
005GH5–1	*Flavobacterium* sp. OSR005	OSR005_04227	W	2D	I
005GH5–2	*Flavobacterium* sp. OSR005	OSR005_03871	G (Gly)	2D	II
*Bo*GH5A	*Bacteroides ovatus*	BACOVA_02653	W	2D	III
*Bo*W252A	*B. ovatus*	BACOVA_02653	A (Ala)	2D	III
*Bo*W252G	*B. ovatus*	BACOVA_02653	G	2D	III
*Cj*GH5	*Cellvibrio japonicus*	CJA_3010	W	2D	I
*Pae*GH5	*Paenibacillus* sp. Root144	2644426200^*^	H (His)	1	NA

^*^denotes the IMG gene accession deposited in the IMG/JGI database.

The candidate *Flavobacterium* XyGUL combined elements of XyG-utilising genes previously identified *in B. ovatus* [54]*, C. japonicus* [21, 22], and *Xanthomonas* spp. [20], the latter including the recently identified O-acetylesterase (CE20) (Fig. 2C & D). GH39, an enzyme predicted to hydrolyse the Xyl(α*1–2*)Araf linkage found in *Solanaceous* plants such as tomato, was only present in *Flavobacterium* spp. (Fig. 2D). In both *Flavobacterium* spp., the TBDT-SusD-like pair, hereafter referred to XusCD, were among the most differently synthesised proteins detected during growth on XyG (Fig. 2B, Tables S5 & S6), supporting their use as markers for in situ utilisation of this hemicellulose. In contrast to XyGUL in *Bacteroides* spp. [55], the *Flavobacterium* XyGUL retains high conservation and synteny across all plant-associated strains analysed (Fig. 2C), with no rearrangements and only few instances of gene insertions. These data suggest *Flavobacterium* spp. harbour a specialised XyGUL for efficient growth on XyG.

### XyGUL encoded proteins are essential for efficient growth on XyG in *F. johnsoniae*

**Figure 3:**
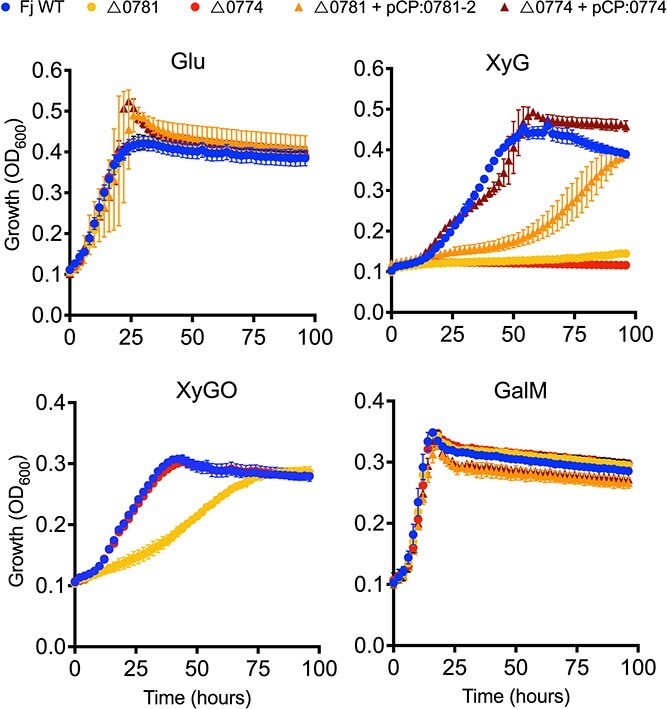
**Genetic basis of xyloglucan utilisation in *Flavobacterium johnsoniae*.** The wild type (circles), the outer membrane GH5_4 endoxyloglucanse (Δ*0774*) mutant (circles), the outer membrane TonB-dependent transporter and cognate lipoprotein (Δ*0781–2, xusCD*) mutant (circles) were grown on either glucose, XyG, XyGO, or GalM as the sole C and energy source. Both mutants were complemented with their respective native genes, *fjoh_0774* (triangles) and *fjoh_0781–2* (*xusCD*, triangles). Growth assays were performed in triplicate and error bars denote the standard deviation from the mean.

To determine the in vivo contribution of XyGUL encoded proteins towards growth on XyG, two knockout strains of *F. johnsoniae* were generated. The first carried a deletion of *fjoh_0774*, encoding the GH5 enzyme predicted to initiate depolymerisation of the XyG polysaccharide. The second mutant carried a deletion of *fjoh_0781–2* encoding the XusCD system predicted to be required for oligosaccharide uptake (Fig. 2D). The isogenic wild-type parent and both mutant strains grew comparably on either glucose or GalM. However Δ*0774* was unable to grow on XyG and the growth of the Δ*0781–2* mutant was significantly curtailed (Fig. 3). These data are consistent with the proteomics analysis (Fig. 2B), demonstrating XyGUL is essential for growth on XyG. These data are also consistent with the “natural” gene knockout strain *Flavobacterium* sp. F52, which lacks the gene encoding the GH5_4 in an otherwise complete XyGUL (Fig. 2A). Complementation of each mutant with an *in trans* copy of the respective gene(s) restored their ability to grow on XyG (Fig. 3). As expected, the Δ*0774* mutant, which lacked the outer membrane initiator enzyme *Fj*GH5, was capable of growth on commercially synthesised XyGOs (Fig. 3). Δ*0781–2* unexpectedly grew on XyGOs, albeit at a slower rate, in contrast to its phenotype on XyG, suggesting *Fj*GH5 and XusCD may interact to coordinate hydrolysis of the polysaccharide backbone.

**Figure 4:**
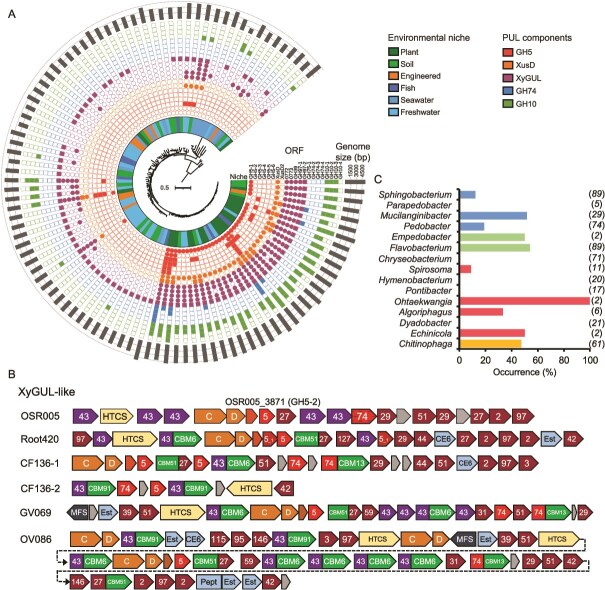
**The occurrence and diversity of GH5_4 homologs in terrestrial *Bacteroidota* species.** (**A**) Phylogenomic analysis of our previously generated multi-loci maximum-likelihood consensus tree, inferred from the comparison of 10 housekeeping and core genes present in 102 *Flavobacterium* isolates (43). The presence (filled symbol) or absence (hollow symbol) of CAZyme ORFs associated with PUL are displayed, as well as the genome size of each isolate (outer ring). The inner ring denotes the environmental niche from which the genome was isolated. (**B**) Selected PUL (XyGUL-like) containing GH5–3 - GH5–6 homologs identified in *Flavobacterium* spp. demonstrating diverse genomic organisation. Numbers denote glycoside hydrolase family predictions. Abbreviations: Est, esterase; Pept, peptidase; CBM, carbohydrate binding module; MFS, major facilitator superfamily transporter; HTCS, hybrid two component sensor. (**C**) The prevalence of GH5_4 homologs in the genomes from different genera within the phylum Bacteroidota, determined through BLASTP (cut off, e^−40^). The number of genomes screened per genus is given in the parentheses. Colours denote the associated class (taxonomic rank).

### Hemicellulose utilisation machinery is enriched in plant-associated bacteroidota

We investigated if XyG utilisation in *Flavobacterium* is an adaptation to life in the plant microbiome by analysing our previous database representing *Flavobacterium* spp. isolated from distinct ecological niches [43]. In addition to searching for GH5_4 homologs, we also searched for homologs related to other XyGUL components and candidate GH10 endoxylanases (pfam00331) required to hydrolyse xylan [56], another hemicellulose root exudate [13]. Open reading frames (ORFs) encoding XyGUL components and GH10 homologs were more prevalent among plant-associated and closely related strains (Fig. 4A). Several plant-associated *Flavobacterium* strains possessed up to six closely related GH5_4 homologs. The most prevalent were the canonical GH5–1 forms found in the XyGUL. GH5–3 - GH5–6 were never located within a XyGUL. GH5–3, the second most prevalent form, was colocalised in a distinct “XyGUL-like” PUL (Fig. 4B). XyGUL-like clusters are present in fewer *Flavobacterium* genomes and have far less gene synteny and conservation than XyGUL, encoding a diverse set of exo-acting GHs, distinct SusCD-like systems, and in some cases a GH74 homolog similar to the endoxyloglucanase recently shown to be functional in *Xanthomonas* spp. [20].

We expanded our search to the wider *Bacteroidota* phylum, encompassing *Chitinophagaceae*, *Sphingobacteraceae, Flavobacteriaceae*, and *Cytophagaceae* (Table S3). Genomes were restricted to those retrieved from terrestrial environments, i.e., soil and plant. Both inter- and intra-genus variation in the occurrence of GH5_4 homologs in the genomes of *Bacteroidota* spp. was detected (Fig. 4C). The highest percentage of genomes possessing GH5_4 homologs belonged to *Flavobacterium* (54%), with almost all plant-associated strains possessing XyGUL. Next was *Mucilaginibacter* (51%) and *Chitinophaga* (46%). Despite belonging to the family *Flavobacteriaceae*, *Chryseobacterium* harboured no GH5_4 homologs, consistent with this genus only responding to simple C additions (Fig. S1). No GH5_4 homologs were found in the genomes of *Pontibacter* and *Hymenobacterium*. *C. pinenesis* DSM2558, which cannot efficiently grow on XyG [27], lacks genes encoding GH5_4 or XyGUL components. Despite identifying GH5_4 homologs in several plant-associated bacteroidota, PUL organisation differed from the *Flavobacterium* XyGUL (Fig. S3), more closely resembling the organisation and features of the XyGUL-like clusters found in *Flavobacterium* spp. (Fig. 4B), which was not induced during growth on XyG in OSR005 (Table S6). Phylogenetics confirmed GH5_4 homologs identified in other genera affiliated with *Bacteroidota* were separated into various subclades like *Flavobacterium* (Fig. S4).

### GH5_4 subclade 2D has radiated in soil and plant microbiomes

**Figure 5:**
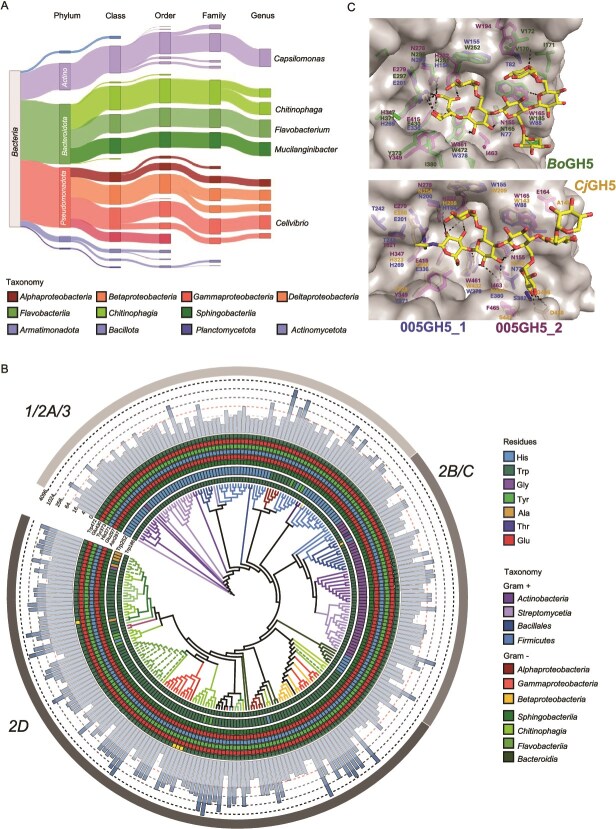
**Distribution of GH5_4 homologs in soil- and plant-associated metagenomes.** (**A**) Taxonomic classification and relative abundance of ORFs retrieved from bulk soil, rhizosphere and root endosphere. ORFs were identified through BLASTp against the CAZYdb (**B**) Reconstructed phylogeny (maximum likelihood method, bootstrap 1000) of GH5_4 homologs in the CAZyme database that best represent the ORFs retrieved from the metagenomes. The amino acid present at each of the key residue sites experimentally determined in previous studies are presented as coloured rings. The outer bar plots represent the overall gene abundance across all metagenomes. Branches are coloured based on their taxonomic classification at the class level. The outer ring represents the GH5_4 clades (1, 2, or 3) previously identified by [56]. (**C**) Key residues for activity in *Bo*GH5A (pdb: 3zmr) and *Cj*GH5 (pdb: 5oyd) and the corresponding conserved residues in the modelled 005GH5–1 and 005GH5–2 were depicted using PyMOL (v2.6).

The GH5_4 family has recently been structured into three main clades (named 1, 2, 3) and subclades, with *Bo*GH5A and *Cj*GH5d belonging to subclade 2D [56]. Given the high prevalence of GH5_4 homologs in plant-associated bacteroidota, we performed BLASTP on over 700 plant/soil metagenomes deposited in the IMG/JGI database (Table S4). Two GH5 sequences were used as queries: *Fj*GH5 (Fjoh_0774) and a GH5_4 from *Paenibacillus* sp. Root144 [57], representing clade 1. All environmental ORFs retrieved (n = 7636) were locally aligned (BLASTP) against GH5 in the CAZYdb (n = 1123) [25]. 7136 environmental ORFs mapped to 254 CAZYdb ORFs, specifically the GH5_4 subfamily. Homologs related to *Bacteroidota* (N = 39 150) and *Proteobacteria* (n = 39 031) constituted much of the diversity found in soil (Fig. 5A). At the genus-level, homologs related to *Capsulimonas* (*Actinomycetota*, n = 11 783) and *Flavobacterium* (n = 11 232) were the most abundant, followed by *Cellvibrio* (n = 8700), *Mucilaginibacter* (n = 8697), and members of the family *Chitinophagaceae* (*Pseudobacter*; n = 7967, *Chitinophaga*; n = 5516).

Phylogenetic analysis revealed subclade 2D, which contains *Fj*GH5 and *Cj*GH5, represented the bulk of GH5_4 abundance in soil and plant microbiomes (Fig. 5B). Alignment of the eight key residues required for binding, coordination and hydrolysis of the polysaccharide were highly conserved, apart from Trp252 (*Bo*GH5 as reference, Trp209 in *Cj*GH5) [56]. Although Trp252 is highly conserved in most subclade 2D GH5_4 homologs, this residue was predominantly substituted with either His or Gly in clades 1, 3, and subclades 2A, 2B, and 2C (Fig. 5B). Trp252 is conserved in almost all *Flavobacterium* clade 2D homologs, including *Fj*GH5, and the GH5_4 enzyme encoded by OSR005_04227 in *Flavobacterium* sp. OSR005 (Fig. 2), hereafter termed 005GH5–1. However, several *Flavobacterium* clade 2D GH5_4 (labelled GH5–3 – GH5–6 in Fig. 4A), which were not located in XyGUL, also carried a mutation at this site. Typically, Trp was substituted with either Gly or Ala (Figs. S2 and S4). OSR005 also carried this second GH5_4 form (005GH5–2) harbouring the Ala mutation and was not detected during growth on XyG (Table S6). Based on structural homology modelling and previous structural data for *Bo*GH5A and *Cj*GH5d [22, 55], Trp252/209 forms a stacking interaction with the xylose residue occupying the −2 glucose position in XX**(X)**G-type saccharides, such as tamarind XyG (Fig. 5C; Fig. S5). Enzymes from all clades demonstrate promiscuous activity towards a range of polysaccharides, including, mannan, xylan, and linear glucans (lichenin) [56]. A notable exception is *Cj*GH5 that belongs to subclade 2D. Clade 1 and 3 homologs that display greatest promiscuous activity have a markedly different surface in this region, typically possessing a cavity. Alignment of modelled 005GH5–1 and 005GH5–2 revealed this residue was absent in the latter and likely results in a cavity reducing any interaction with the third xylose (Fig. S6).

### Activity towards XyG in GH5_4 subclade 2D homologs is mediated by a key residue

**Figure 6:**
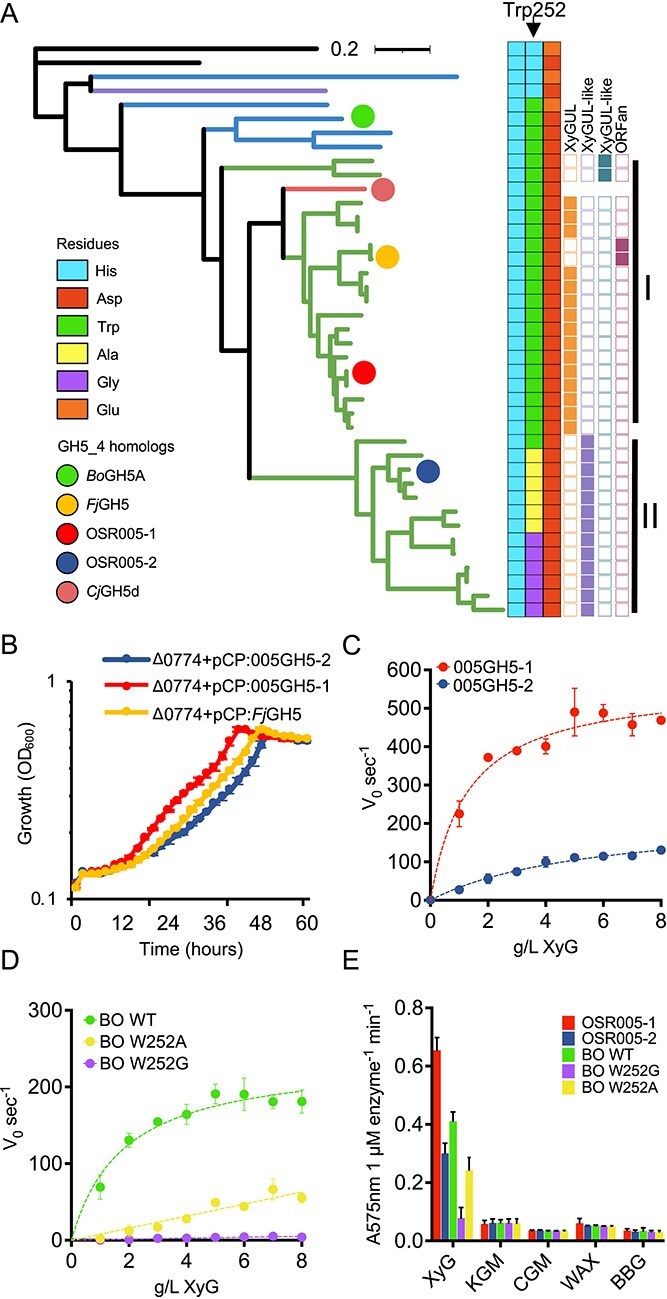
**Characterisation of GH5_4 homologs in *Flavobacterium* species.** (**A**) Phylogenetic reconstruction of GH5_4 homologs identified in *Flavobacterium* spp. alongside those previously characterised, showing the variable Trp252 residue (*Bo*GH5A) and each adjacent amino acid residue. The genomic localisation of the GH5_4 homologs is given in columns to the right of the residues. The Trp-containing forms in *Flavobacterium* are almost exclusively associated with XyGUL. I and II represent the identified type I and type II *Flavobacterium* GH5_4 homologs. (**B**) Growth (n = 3) of the *F. johnsoniae* Δ*0774* mutant complemented with either its native gene homolog or the two *Flavobacterium* sp. OSR005 GH5_4 homologs (005GH5–1, 005GH5–2). Enzyme kinetics for DNSA assays were performed to determine endoxyloglucanase activity (tamarind XyG) of purified recombinant GH5_4 homologs from *Flavobacterium* sp. OSR005 (**C**) and *Bo*GH5A wild type (WT), W252A and W252G variants (**D**). These five recombinant GH5_4 enzymes were also tested for activity against Konjac-glucomannan (KGM), carob-galactomannan (CGM), wheat arabinoxylan (WAX), and barley beta-glucan (BBG) (**E**). Reactions (n = 3) were stopped after 10 min and reducing sugar ends were quantified (Abs575 nm) and normalised to 1 μM enzyme per min. Error bars denote standard deviation.

Closer inspection of *Flavobacterium* GH5_4 homologs revealed two groups nested within subclade 2D, which we refer to as Type I and Type II (Figs. 6A and S2). Type I clustered with the XyG-specific *Cj*GH5, whereas Type II formed a distinct group. All GH5_4 homologs with Trp replaced by either Ala or Gly were found in the Type II cluster. All Type I were in XyGUL whereas Type II were not, being typically located in the distinct XyGUL-like clusters (Figs. 4B and S2).

To determine if Type II GH5_4 homologs were functional, we complemented the *F. johnsoniae* Δ*0774* mutant with the genes encoding 005GH5–1 (Type I) and 005GH5–2 (Type I) expressed from the constitutive *ompAFj* promoter. Both 005GH5–1 and 005GH5–2 restored the ability of Δ*0774* to grow on XyG as the sole C source, with the 005GH5–1 strain showing a greater initial growth rate and 005GH5–2 the slowest (Fig. 6B). To test if the lower growth rate observed for 005GH5–2, which carries the W252A substitution, was due to a lower enzyme activity, we purified recombinant OSR005–1, 005GH5–2, and the archetypal *Bo*GH5A following heterologous over-production in *E. coli*. Recombinant 005GH5–1 had a significantly greater turnover rate (*K*_cat_ = 566.2 min^−1^) than recombinant 005GH5–2 (*K*_cat_ = 223.5 min^−1^) and a lower *K*_m_ (OSR005–1 = 1.3 mg ml^−1^, OSR005–2 = 5.7 mg ml^−1^) (Fig. 6C). Recombinant *Bo*GH5A modified with either W252A or W252G substitutions replicated this dramatic reduction in endoxyloglucanase activity (Fig. 6D). Neither OSR005–1, OSR005–2, *Bo*GH5A, *Bo*W252A, nor *Bo*W252G conveyed substrate promiscuity towards other glycans typically found in the plant microbiome (Fig. 6E).

## Discussion

High-throughput sequencing has revealed taxa related to *Bacteroidota*, particularly *Flavobacterium*, are highly enriched in the plant microbiome [32–34]. However, *Bacteroidota* spp. are typically not enriched when soil rhizosphere samples are supplemented with structurally simple C substrates [58–62]. Here, we reveal *Flavobacterium* spp. lack the ability to compete for simple C and instead efficiently grow using the plant hemicellulose XyG, enabled through the possession of XyGUL. This metabolism may provide bacteroidota with a competitive advantage when invading and persisting in the plant microbiome, facilitated through resource diversification [5, 23]. The high prevalence of XyGUL in plant-associated genomes and low prevalence in those retrieved from other environmental niches, such as seawater, further suggests XyG utilisation as a strategy to succeed in the plant microbiome, similar to their organophosphorus utilisation [43] and predicted plant pectin degradation capabilities [63, 64].

Except for *Xanthomonas* spp. [20], endo-acting xyloglucanases (GH5_4 and GH74) found in the genomes of non-*Bacteroidota* are rarely co-localised in organised XyGUL or clusters bearing resemblance. Notable exceptions included individual strains of *Massilia* and *Pelomonas.* Furthermore, TBDTs are absent in Gram-positive bacteria that otherwise encode extracellular endoxyloglucanases. The functionality of these XyGUL-like clusters is hard to predict as ORF content and synteny differs from *Flavobacterium* or *Xanthomonas* XyGUL [20]. The presence of a XyGUL-like cluster in *Flavobacterium* sp. OSR005 containing a predicted GH74 and 005GH5–2, which is otherwise not induced by XyG, further demonstrates the need for experimental validation to support functional prediction. *C. japonicus* encodes several endoxyloglucanases that are scattered around the genome and away from the gene cluster encoding XusC and auxiliary enzymes [21, 22], therefore a strict XyGUL is not essential for XyG utilisation. Although taxa affiliated with *Gammaproteobacteria*, such as *Cellvibrio* and *Xanthomonas*, possess endo-acting xyloglucanases, and TBDTs, akin to XusC [20, 21], they lack the surface-exposed glycan binding domain (XusD) identified in this study. Our simple soil enrichment suggests possession of a conserved XyGUL containing XusCD, a XyG-specific GH5_4, and auxiliary enzymes, does increase the competitive ability of *Flavobacterium* to capture these complex exudates [65], consistent with the ecological function of these transporters in marine and gut microbiomes [66–68]. The lack of high-affinity ABC transporters in plant *Bacteroidota* spp. also suggests they cannot compete for the full range of diverse simple C substrates produced by plant roots, also supported by soil enrichments.

Terrestrial bacteroidota can utilise other complex C substrates, including pectin [64], alternative plant cell wall components [17, 27, 28], and fungal polysaccharides [29, 69]. Together with our data, these observations support a model whereby complex C is the preferential nutrient and energy source for bacteroidota in soil and plant microbiomes. The domestication of agricultural crops is driving a significant loss of various key microbiota, including taxa affiliated with *Bacteroidota* [9, 37], hypothesised to be a consequence of changes in crop root exudation profiles with a relative increase in the ratio of simple: complex C [8]. This reduction in beneficial microbes, such as *Flavobacteraceae* and *Chitinophagaceae*, may have negative impacts on agricultural soil health [70] and the plants ability to suppress pathogens [35–38, 71, 72]. Interestingly, the relative abundance of genes encoding XyGUL components, such as GH5, GH31, GH3, and GH95 were also significantly higher in metagenomes sampled from healthy versus diseased pepper plants, when challenged with *Fusarium* [72]. Collectively, these studies and ours highlight a possible link between plant bacteroidota, complex C utilisation, and plant disease suppression. We propose, future research should focus on explicitly linking the connection between complex exudation and the assemblage of bacteroidota in the plant microbiome in the context of crop domestication and host disease. These studies are essential to better understand the drivers of *Bacteroidota* assemblage and host–microbe interactions in the plant microbiome [37]. Deployment of *Bacteroidota* spp. into agricultural systems through inoculation is tempting, however efforts using this approach rarely achieve their desired results in the real world [73]. Investigating the specific plant genetic drivers underpinning bacteroidota recruitment, including landraces and wild progenitor genotypes, would guide future crop breeding trials to help reverse the loss of the beneficial microorganisms in agriculture [37].

Given the proposed importance of plant polysaccharides in soil aggregation and the long-term storage of C [3, 6], degradation of these molecules may represent a significant and relatively overlooked cog in the global C cycle. The comparatively efficient utilisation of glycans by *Bacteroidota* relative to non-*Bacteroidota*, as observed in marine systems [53, 65, 74], may therefore have consequences for the microbial C pump [3], which can be altered by changes in bacterial C use efficiency [75, 76]. Microbial polysaccharides also represent a major fraction of recalcitrant or “stabilised” C in soil, a fraction which is vulnerable to microbial attack in response to a climate-induced influx of labile C or changes in land-use intensity [3, 4, 75, 77]. Whether shifts in the abundance and diversity of *Bacteroidota*, which are known to be good indicators of soil health [8, 70], could influence this key step in the terrestrial global C cycle warrants further investigation.

The lack of XyGUL in certain genera related to *Bacteroidota*, (e.g. *Chryseobacterium*) coupled with a 10–60% occurrence of GH5_4 homologs in other *Bacteroidota* genera, suggests some level of functional partitioning within this phylum. Indeed, *Chryseobacterium* spp. possess an enhanced capability to degrade microbial polysaccharides associated with Gram-positive peptidoglycan compared to *F. johnsoniae* or *Sphingobacterium* sp. [78]. *C. pinensis* also lacks the ability to utilise XyG despite its capability to grow on other hemicelluloses and fungal glycans [27–29, 79] and our comparative genomics confirmed this bacterium lacks a GH5_4 homolog. Hence, whereas bacteroidota invading and persisting in the rhizosphere likely specialise in broad complex C utilisation, metabolic heterogeneity for specific complex C substrates exists within this phylum.

Our data reveals subclade 2D of the GH5_4 has radiated in soil microbiomes and is the dominant form, in contrast with the abundant forms found in engineered systems or animal guts [56]. Clade 2D carried a distinct mutation at Trp252 (position in *Bo*GH5A), which is typically Gly, Ala, or His in clades 1 and 3. Clade 3 GH5_4 enzymes possess high activity towards multiple polysaccharides in addition to xyloglucan, in contrast to clade 2D homologs produced by *C. japonicus* (*Cj*GH5d, *Cj*GH5e, *Cj*GH5f) [22, 79, 80]. Hence, the presence of clade 1 and 3 GH5_4 enzymes in *Actinomycetota* and *Bacillota* may reflect a trade off whereby these bacteria carry fewer CAZymes with greater individual substrate ranges, relative to *Bacteroidota,* to scavenge complex C molecules in bulk soil away from plant roots [81, 82]. Enzyme specificity versus promiscuity is however likely driven by many more mutations that influence active site architecture through alterations in secondary structure [83]. This may explain why mutation of Trp252 *Bo*GH5A did not broaden its substrate range.

For gut *Bacteroidota* members, distinct PUL are required to degrade simple and complex arabinoxylans, which are differentially regulated in response to these different forms of the polysaccharide [23, 71]. A similar phenomenon may explain the existence of Type II GH5_4 homologs, which carry a mutation in one of the eight key residues, and are typically found in PUL that significantly differ from the conserved *Flavobacterium* XyGUL. Indeed, XyG is often part of a larger polysaccharide exudate complex, which includes pectin and xylan complexes [15]. These complex Type II-harbouring PUL may therefore represent specialisation in utilising either non-exudate plant polysaccharides [10] or more complex forms released by plant roots [15].

In summary, we identified a highly conserved XyGUL among plant-associated *Flavobacterium*. Given the emergent knowledge that most plants, including globally important crop species, exude significant quantities of XyG, we propose this hemicellulose is an important C source for plant-associated *Flavobacterium*, enabling their invasion and persistence in a highly competitive plant microbiome.

## Supplementary Material

XyG24_SuppInfo_ISME_wraf022

XyG24_Supplementary_tables_ISME_wraf022

## Data Availability

Publicly available datasets reanalysed in this study are available in the IMG/JGI database. All new DNA and protein sequencing data provided within this manuscript has been uploaded to the appropriate repositories. Proteomics accessions are provided in the materials and methods. DNA sequencing data (16S rRNA gene profiling) has been deposited in the NCBI SRA under the accession PRJNA1173120.
