# Supplementary material for: Metabolism of hemicelluloses by root-associated Bacteroidota species
Source: ISME J. 2025 Feb 6;19(1):wraf022. doi: 10.1093/ismejo/wraf022 (PMC11892949; doi:10.1093/ismejo/wraf022)
Supplement: XyG24_SuppInfo_ISME_wraf022 [file xyg24_suppinfo_isme_wraf022.pdf]

Metabolism of hemicelluloses by root-associated *Bacteroidota* species

Hannah Martin[1+], Lucy A. Rogers[1+], Laila Moushtaq[1], Amanda A. Brindley[2], Polly Forbes[1], Amy R. Quintion[1], Andrew R.J. Murphy[3], Helen Hipperson[2], Tim J. Daniell[2], Didier Ndeh[4], Sam Amsbury[2], Andrew Hitchcock[1,2] and Ian D.E.A. Lidbury[1*]

[1] Molecular Microbiology - Biochemistry and Disease, School of Biosciences, The University of Sheffield, Sheffield, UK

[2] Plants, Photosynthesis and Soil, School of Biosciences, The University of Sheffield, Sheffield, UK

[3] School of Life Sciences, University of Warwick, Coventry, UK

[4] School of Life Sciences, University of Dundee, Dundee, UK

[+]Both authors contributed equally to this study.

***Corresponding author:** I.lidbury@sheffield.ac.uk

Address: Ian Lidbury, School of Biosciences, The University of Sheffield, Western Bank, Sheffield, S10 2TN, South Yorkshire, UK

Running title: Rhizosphere hemicellulose degradation

Contents

Supplementary methods

1. Comparative proteomics of *Flavobacterium* spp.

2. Bacterial genetics

3. Production and purification of recombinant GH5_4 homologs

4. DNA extraction and amplicon sequencing

Supplementary figures

Figure S1. Microbial community profiling (16S rRNA gene) of grassland soil enrichments

Figure S2. Phylogenetic reconstruction of Type I and Type II GH5_4 homologs identified in *Flavobacterium* spp.

Figure S3. XyGUL-like clusters identified in other Bacteroidota spp. based on the possession of GH5_4 homologs.

Figure S4. Phylogenetic reconstruction of the identified GH5_4 homologs found in the genomes of terrestrial Bacteroidota spp.

Figure S5. The molecular basis of protein-XyG binding by BoGH5A

Figure S6. Structural comparison of 005GH51 and 005GH5-2

Figure S7. Enzyme assays using recombinant BoGH5A incubated with XyG

Supplementary Tables (see separate excel file)

Table S1. The occurrence of ORFs containing transporter-related Pfam domains found in plant bacteria.

Table S2. List of primers used in this study

Table S3. Details of the isolates genomes deposited in IMG/JGI that were scrutinised in this study

Table S4. Details of the soil/plant metgenomes deposited in IMG/JGI that were scrutinised in this study

Table S5. Comparative whole-cell proteomics of *Flavobacterium johnsoniae* DSM2064 (UW101) grown on xyloglucan or glucose. Data files were generated using MaxQuant and Perseus

Table S6. Comparative whole-cell proteomics of *Flavobacterium* sp. OSR005 grown on xyloglucan or glucose. Data files were generated using MaxQuant and Perseus

1. Comparative proteomics of *Flavobacterium* spp.

25 mL cell cultures (n=3) grown to an OD_{600} ~0.6-1 were harvested by centrifugation at 3200 x g for 45 min at 4°C. Cells were resuspended in 20 mM Tris-HCl pH 7.8 and re-pelleted at 13000 x g for 5 min at 4°C. Cell lysis was achieved by boiling in 100 µl lithium dodecyl sulphate (LDS) buffer (Expedeon) prior to loading 20 µl onto a 4-20% Bis-Tris sodium dodecyl sulphate (SDS) precast gel (Expedeon). SDS-PAGE was performed with RunBlue SDS Running Buffer (TEO-Tricine) 1X (Expedeon) at 140 V for 5-10 min. Gels were stained with Instant Blue (Expedeon). A single gel band containing all the protein was excised. Gel sections were de-stained with 50 mM ammonium bicarbonate in 50% (v/v) ethanol, dehydrated with 100% ethanol, reduced and alkylated with Tris-2-carboxyethylphosphine (TCEP) and iodoacetamide (IAA), washed with 50 mM ammonium bicarbonate in 50% (v/v) ethanol and dehydrated with 100% ethanol prior to overnight digestion with trypsin. Samples were analysed by nanoLC-ESI-MS/MS using an Ultimate 3000 LC system (Dionex-LC Packings) coupled to an Orbitrap Fusion mass spectrometer (Thermo Scientific, USA) using a 60 min LC separation on a 25 cm column. Resulting tandem mass spectrometry (MS/MS) files were searched against the relevant protein sequence database (*F. johnsoniae* UW101, UP000214645, *Flavobacterium* sp. OSR005 (IMG genome (Taxon) ID: 8103301142) using MaxQuant with default settings and quantification was achieved using Label Free Quantification (LFQ). Statistical analysis and data visualisation of the proteomes was performed in Perseus. The mass spectrometry proteomics data have been deposited to the ProteomeXchange Consortium via the PRIDE partner repository with the dataset identifier PXD053370.

2. Bacterial genetics

Fragments ~1.5 kb in length upstream and downstream of the targeted genes were cloned into the plasmid pYT313 using the HiFi assembly kit (New England Biosciences). Plasmid inserts were verified by Sanger sequencing. The resulting plasmids were transformed into the donor strain *E. coli* S17-1 λpir (S17-1 λpir) and mobilised into *F. johnsoniae* via conjugation: overnight (5 mL) *F. johnsoniae* wild type and pYT313-transformed S17-1 λ*pir* cultures were inoculated (20% v/v) into fresh CYE (5 mL) and incubated for a further 8 h. Cells were pelleted at 1800 x g for 10 min @ 22°C and washed in 1 mL CYE, and a 200 µL donor: recipient (CYE) suspension (1:1) was spotted onto CYE containing $CaCl_2$ (10 mM) and incubated overnight at 28 °C. Biofilms were scraped from the agar surface and resuspended in 1 mL minimal A medium (no C source). Transconjugants were selected by spreading 5 to 100 µL aliquots on CYE containing erythromycin (100 µg mL^{-1}). Colonies were restreaked onto CYE erythromycin and single homologous recombination events were confirmed by PCR prior to overnight growth in CYE followed by plating onto CYE containing 10% (w/v) sucrose to select for a second recombination event resulting in plasmid excision. To identify double homologous recombinants, colonies were replica plated onto CYE containing 10% (w/v) sucrose and CYE containing erythromycin. Erythromycin-sensitive colonies were screened by PCR.

For complementation of the *F. johnsoniae* mutants, both genes and the 300-bp upstream region were cloned into pCP11 using the HiFi assembly kit. The insert was verified by Sanger sequencing and the plasmid was mobilised into DSM2064 via conjugation using S17-1 λ*pir* as the donor strain. The method was identical to that described above for transfer of the suicide plasmid, pYT313, except that 1 mL overnight cultures of donor and recipient were directly washed and resuspended in 200 µL CYE prior to spotting onto CYE containing $CaCl_2$ (10 mM). Cells were scraped from the solid medium and transformants selected by creating a serial dilution (10^{-1} to 10^{-5}) from the cell suspension and spotting 20 µL of each dilution onto CYE containing erythromycin (100 µg mL^{-1}).

3. Production and purification of recombinant GH5_4 homologs

Genes encoding the GH5_4 homologs (Fjoh_0774, BACOVA_02653, OSR005_04227 and OSR005_03871) lacking the N-terminal signal peptide and stop codon were amplified by PCR and ligated into the NdeI and XhoI sites of pET21a. Site-directed mutagenesis of the Trp252 residue in *Bo*GH5A was performed using the QuikChange II Site Directed Mutagenesis (SDM) Kit (Agilent Technologies) according to the manufacturer's protocol.

For production of recombinant proteins, a single colony of *E. coli* BL21 (DE3) transformed with the desired plasmid was inoculated in 5 mL LB broth with 100 µg/mL ampicillin and shaken (220 rpm) at 37 °C overnight (16 h) before transfer to 1 L LB culture (in a 2 L conical flask) supplemented with 100 µg/mL ampicillin. Cultures were shaken at 37 °C at 220 rpm until an optical density at 600 nm (OD_{600}) of ~ 0.6 was reached. Following induction of gene expression with 0.4 mM (final concentration) IPTG, cells were incubated at 18°C overnight for a further 16 h before recovery by centrifugation at 8,000 x *g* for 15 min at 4°C. Pellets were resuspended in 30 mL binding buffer (25 mM HEPES pH 7.4, 1 M NaCl, 5 mM imidazole) and stored at -20 °C until purification. Cells were thawed and lysed by sonication. The lysate was centrifuged at 13,000 *x g* for 15 min at 4 °C and the supernatant was loaded onto a 5 ml chelating Sepharose column charged with nickel (II) sulphate pre-equilibrated with 50 mL of binding buffer. Following washes in binding buffer with increasing concentrations of imidazole, proteins were eluted with 25 mM HEPES pH 7.4, 400 mM Imidazole, 100 mM NaCl. Fractions containing the target protein (as identified by SDS-PAGE) were pooled and concentrated to a volume of 1-2 mL using a Vivaspin centrifuge concentrator (Sartorius) with a 30,000 kDa molecular weight cut off. The concentrated sample was loaded onto a size exclusion chromatography (SEC) (S200 16/60 Cytiva) column equilibrated in 50 mM Tris-HCl, 200 mM NaCl, 10% (w/v) glycerol and protein were separated at a flow rate of 0.5 mL min^{-1}. Purity of peak fractions was analysed by SDS-PAGE and protein was stored at -70°C until required.

4. DNA extraction and amplicon sequencing

Grassland soil samples were collected (June, 2021) from High Bradfield Field Research Station affiliated with the University of Sheffield. Soil cores containing plant roots were collected and roots were removed prior to sieving. After homogenisation, 2g soil (fresh weight) was added to microcosms (n=6) containing 6 mL minimal medium (see main methods for medium composition) supplemented with either 0.3% (w/v) simple C (n=3, glucose, succinate, malate) or complex C (n=3, tamarind XyG, wheat arabinoxylan, konjac glucomannan). 0.5 g (wet weight) soil was instantaneously removed to measure the native microbial community. After 48 h shaking (150 rpm) incubation at 22°C , 0.5 g (wet weight) soil removed from each replicate. In total 12 samples (2 time points X 2 treatments X 3 replicates) were processed for DNA extraction using the POWERSOIL DNA extraction kit (Qiagen). DNA was quantified using BioDrop. PCR was performed using 30 µg of sample template, the 27F-1492R primer pair, and Q5 polymerase (NEB). For time point 0, replicate DNA templates for each treatment were mixed in equimolar amounts prior to PCR. Sequencing was performed on an Oxford Nanopore MINion using the EPI2ME software for QC and taxonomic profiling. As quality control, two samples were also subjected local DIAMOND BLAST against the NCBI 16S RefSeq database and taxonomy was compared. Results showed near perfect congruence therefore MINion outputs were used.

Figure S1. Microbial community profiling (16S rRNA gene) of grassland soil enrichments. Grassland soil (1g), collected from High Bradfield Field Station, Sheffield, was added to minimal medium supplemented with either 0.3% w/v simple C (glucose, succinate, malic acid) or complex C (tamarind XyG, wheat arabinoxylan, glucomannan). For complex C samples, after 48 h (T1), enrichments were sub-cultured (10% v/v) into fresh medium and incubated for a further 48 hours (T2). Taxa was called at the family level, except for Acidobacteroidota and Alphaproteobacteria.

Figure S1

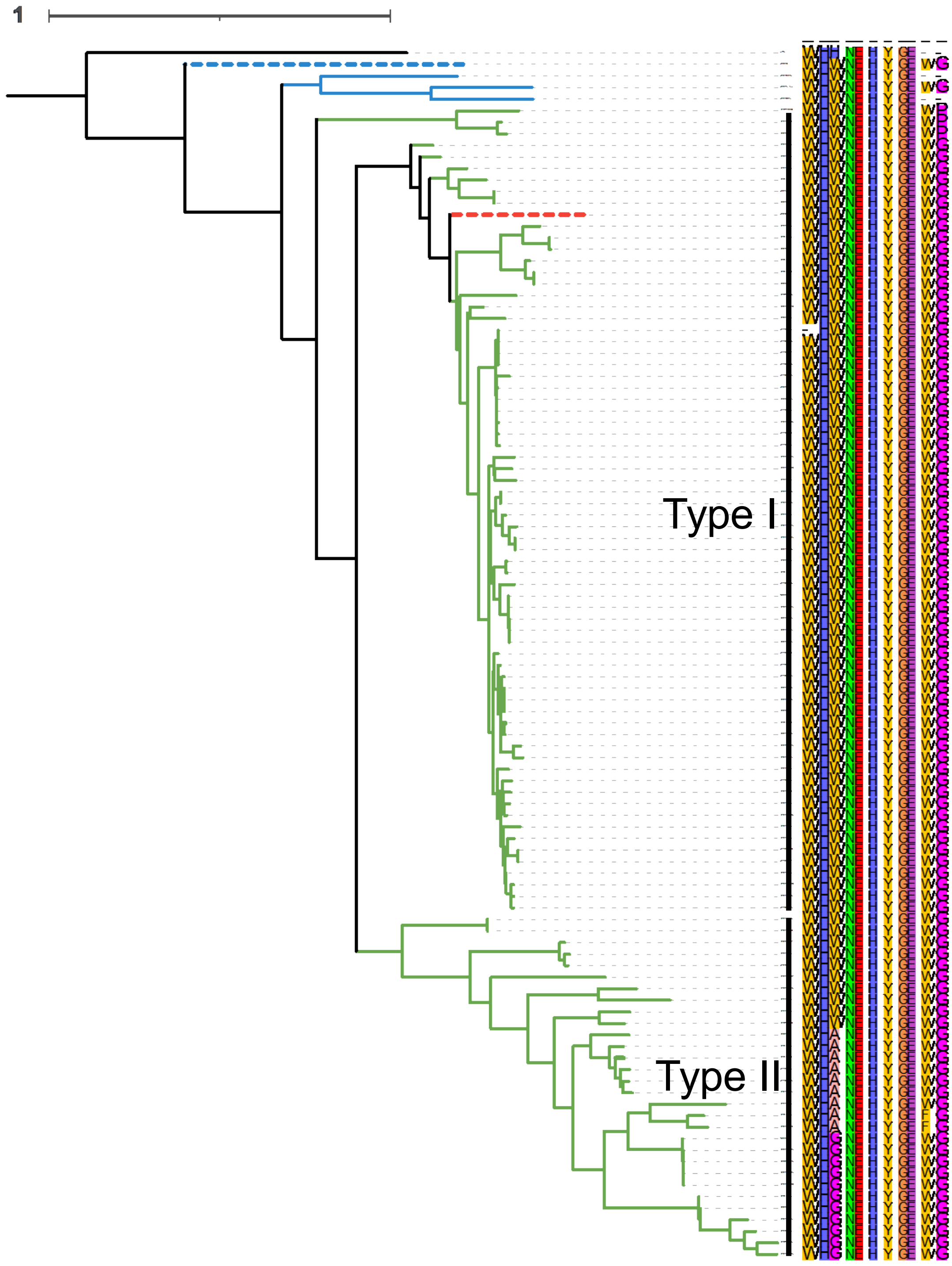

Figure S2. Phylogenetic reconstruction of Type I and Type II GH5_4 homologs identified in *Flavobacterium* spp. Topology was inferred using the maximum-likelihood consensus method (bootstrap = 1000), and the most appropriate parsimony model was selected using IQTREE. Previously characterised homologs from *C. japonicus* (red dotted line) and *B. ovatus* showing the variable Trp252 residue (*Bo*GH5A) and either adjacent amino acid.

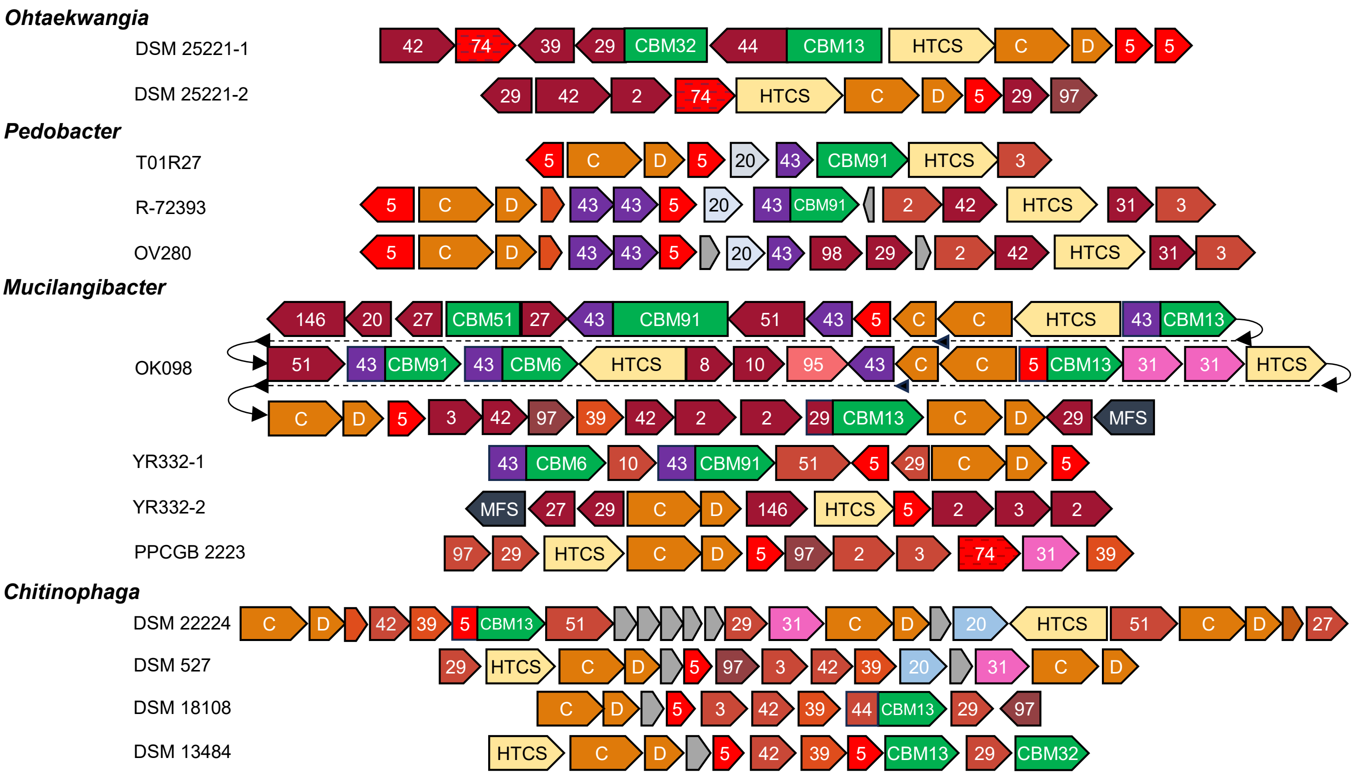

Figure S3. XyGUL-like clusters identified in other Bacteroidota spp. based on the possession of GH5_4 homologs. *Flavobacterium* spp. demonstrating diverse genomic organisation. Numbers denote glycoside hydrolase family predictions except for 20 (sky blue), which denotes a carbohydrate esterase family enzyme. Abbreviations: CBM, carbohydrate binding module, HTCS, hybrid two component sensor.

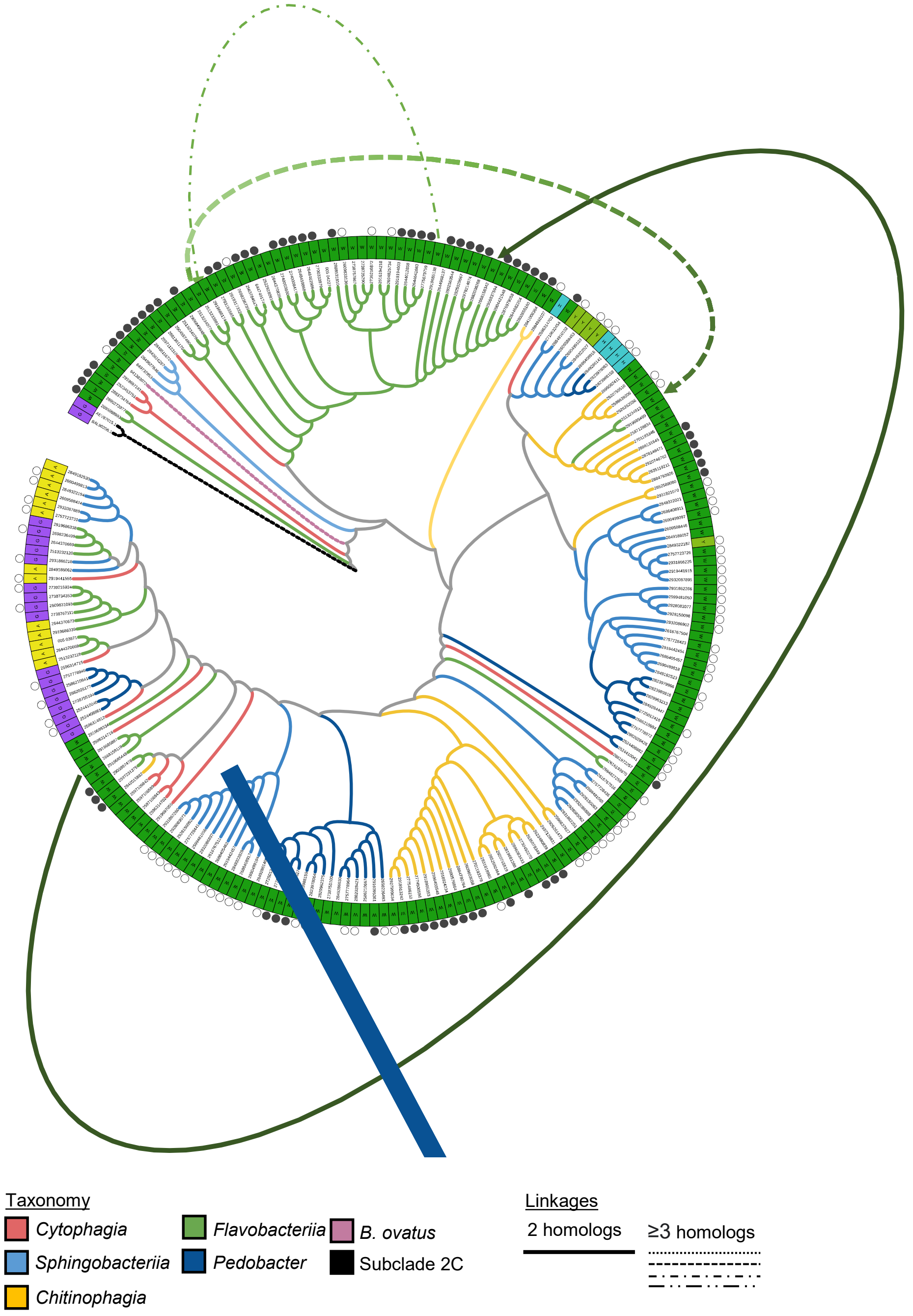

Figure S4. Phylogenetic reconstruction of the identified GH5_4 homologs found in the genomes of terrestrial Bacteroidota spp. Topology was inferred using the maximum-likelihood consensus method (bootstrap = 1000), and the most appropriate parsimony model was selected using IQTREE. Linkages between homologs identified from the same genome depicted by the inverted CICRUS plot. Linkage direction has no biological relevance, only to help trace individual connections. Where linkages were omitted for clarity, black dots denote cases where a single homolog is present in the genome (i.e. *F. johnsoniae*), whilst white dots denote homologs where multiple forms were identified in one genome (i.e., OSR005). Note that some genomes contain up to seven GH5_4 homologs. Linkage colours and tree branches correspond to a specific taxonomic classification (see legend) and dashed or dotted linkages represent three or more homologs identified within a single genome. There are no instances were GH5_4 homologs carrying the Ala or Gly residue were the single homology in a genome.

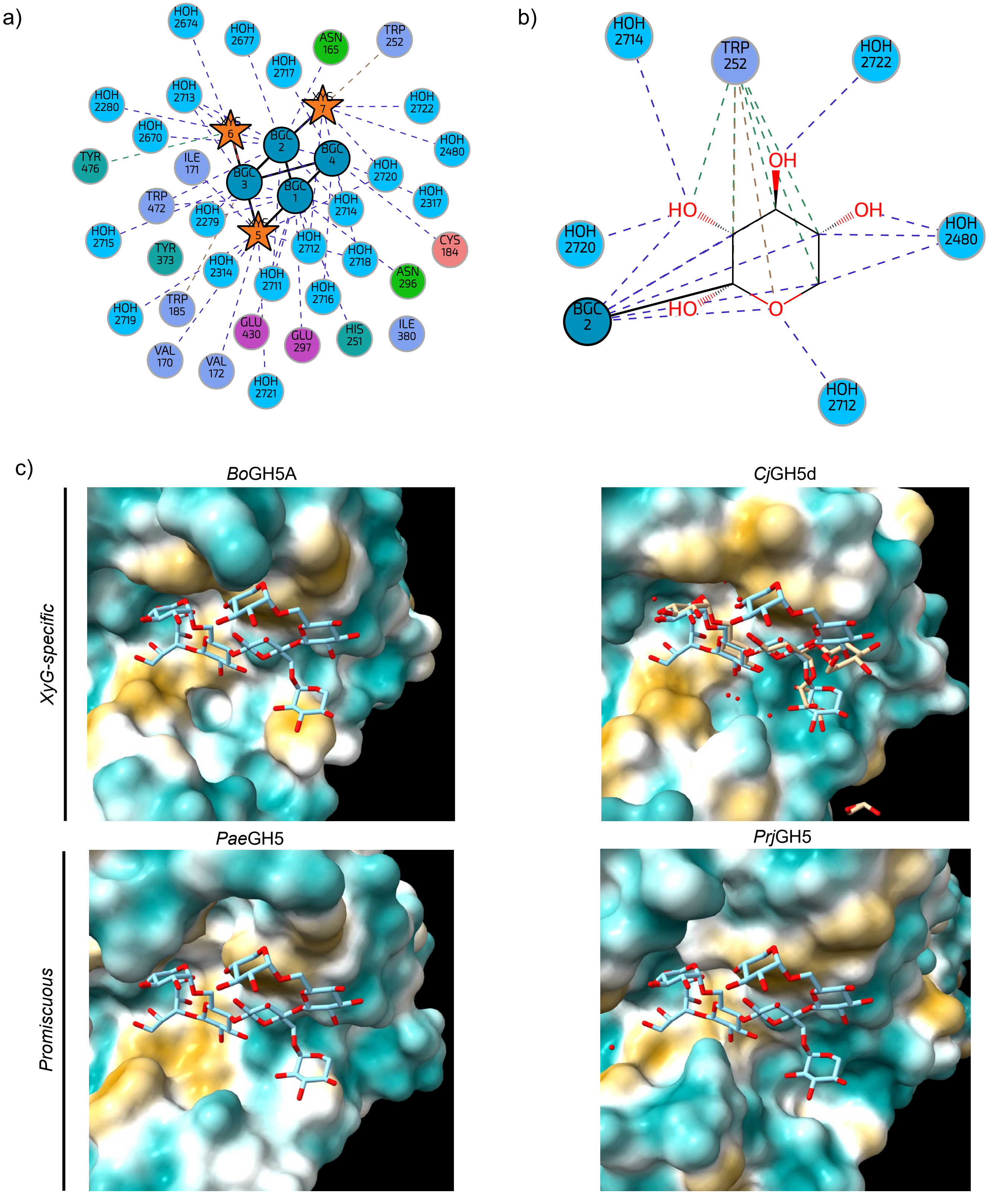

Figure S4. The molecular basis of protein-XyG binding by BoGH5A. Molecular interactions between BoGH5A and XyG **(a)** and the third xylose side branch found in a typical XXXG motif **(b)**, including those occurring with Trp252 (green dashes). Interactions were calculated using Arpeggio that is available for use on the PDBe web server, using the carbohydrate polymer function in ligand and environment. c) The cleft (0-4 subsites) of XyG-specific and promiscuous GH5_4 enzymes with XyG oligosaccharide modelled.

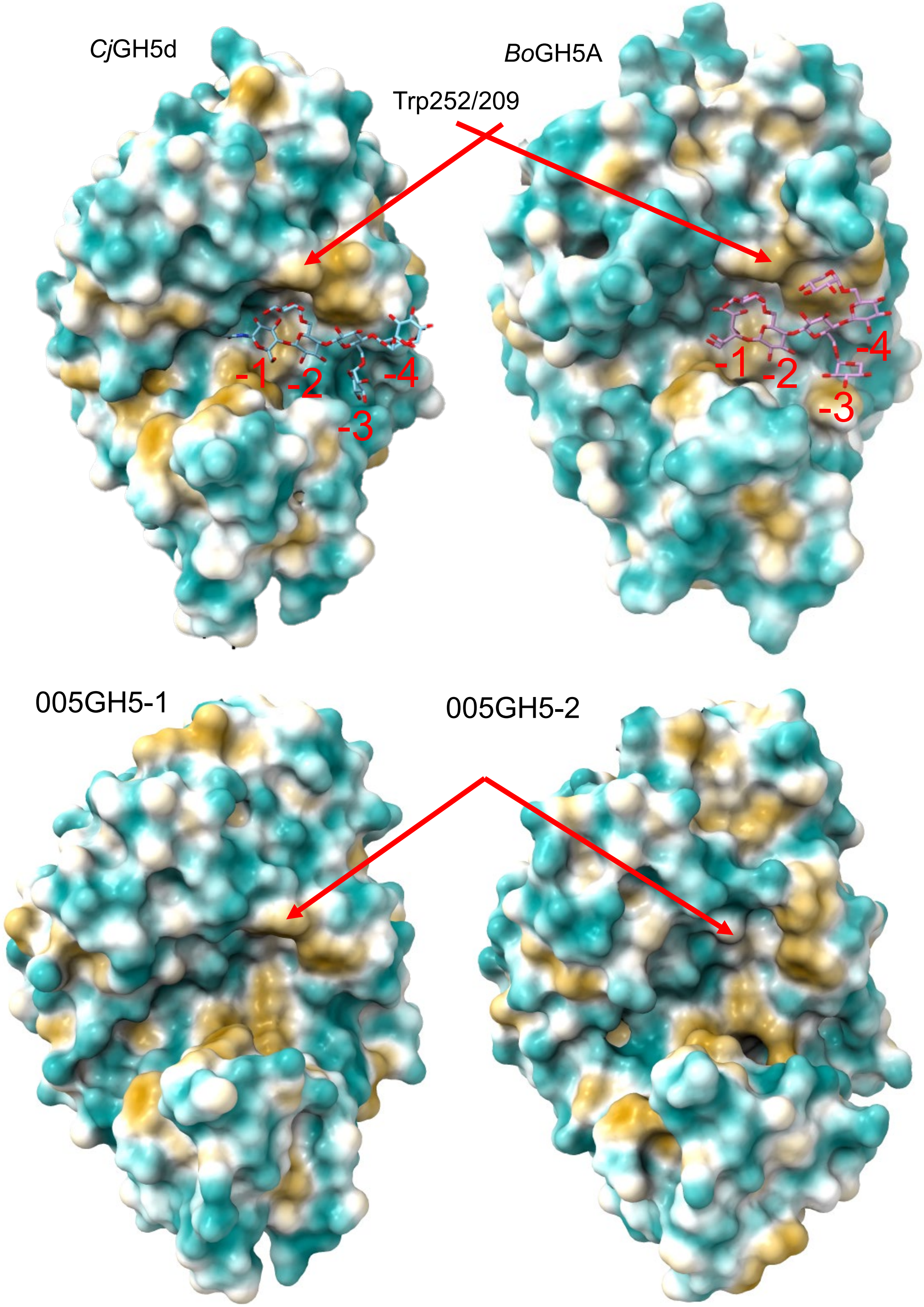

Figure S5. Structural comparison of 005GH51 and 005GH5-2. *Cj*GH5d (pdb: 5oyd) and BoGH5A (pdb: 3zmr) structures depicting surface hydrophobicity determined by X-Ray crystallography modelled with XyG bound visualising the stacking interaction between the third xylose side branch found in a typical XXXG motif, including those occurring with Trp252/209 at the -2 subsite. Alphafold2 generated models of 005GH5-1 and 005GH5-2. Arrows indicate the Trp residue that is substituted with Ala in 005GH5-2. Enzyme and growth assays were performed in triplicate and error bars denote the standard deviation from the mean.

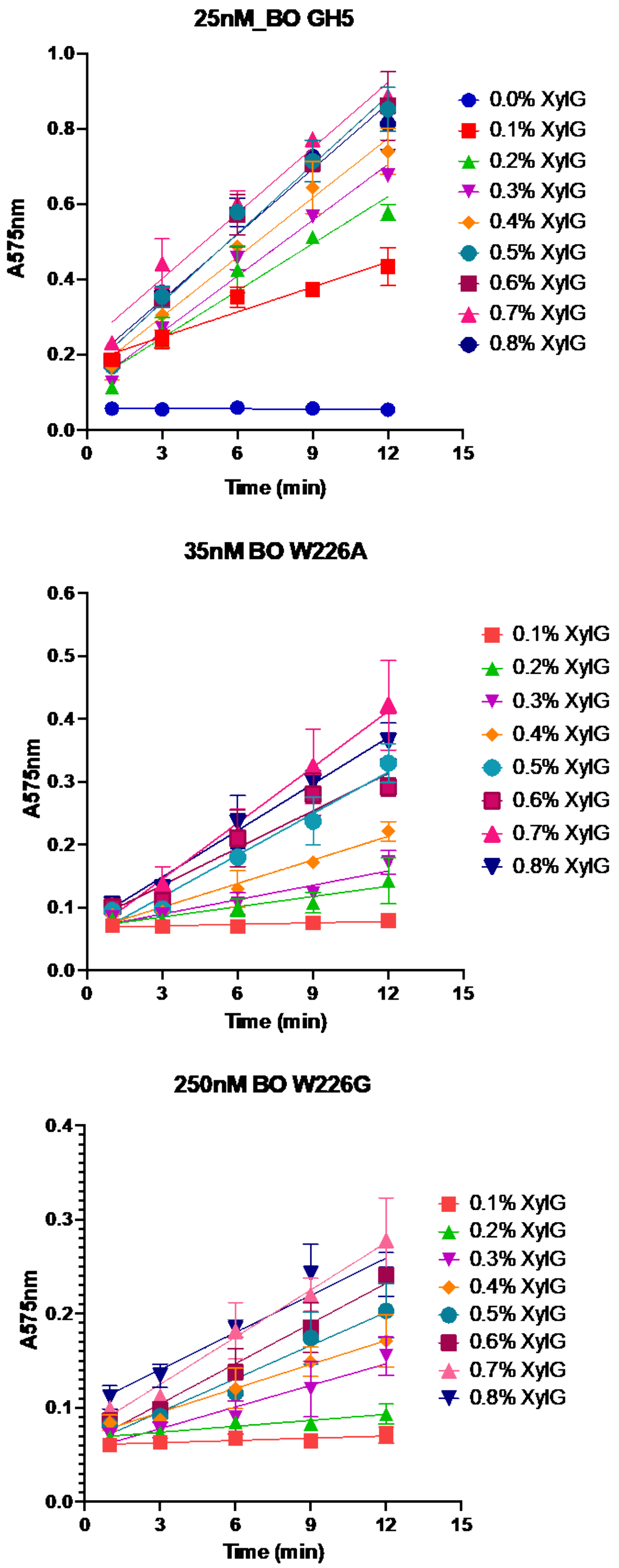

Figure S7. Enzyme assays using recombinant BoGH5A incubated with XyG. Initial enzyme reaction velocity of *Bo*GH4A wild type and mutants were quantified using reducing sugar assays (DNSA) (n=3) against an increasing concentration of XyG. For mutants, higher conentrations of enzyme were used in order to accurately quantify the initial velocity (V_o).
